# Medial Condyle Fracture of the Distal Humerus in an Elderly Patient With Fishtail Deformity and Lateral Condyle Nonunion: A Case Report

**DOI:** 10.7759/cureus.39289

**Published:** 2023-05-21

**Authors:** Hiroki Kawamata, Taku Hatta, Atsushi Takahashi, Satoshi Tateda, Mika Abe

**Affiliations:** 1 Department of Orthopedic Surgery, Sports Clinic Ishinomaki, Ishinomaki, JPN; 2 Department of Orthopedic Surgery, Japanese Red Cross Ishinomaki Hospital, Ishinomaki, JPN

**Keywords:** nonunion, lateral condyle, total elbow arthroplasty, fishtail deformity, medial condyle fracture

## Abstract

A medial condyle fracture of the humerus with preexisting fishtail deformity and lateral condyle nonunion is very rare, and there have been few reports describing favorable treatment options. We herein report the case of an 83-year-old woman who sustained a medial condyle fracture of her elbow with a comorbidity of long-lasting limited elbow motion with a history of elbow trauma in childhood. After conservative treatment with casting for four weeks, unstable medial condyle fracture in the presence of fishtail deformity and lateral condyle nonunion remained. Due to persistent pain, the patient underwent surgical treatment with semiconstrained total elbow arthroplasty (TEA) through the triceps-on approach. At the 12-month follow-up examination, the patient had no pain and achieved satisfactory functional outcomes. This case report demonstrated the efficacy of TEA for deteriorated stability due to bilateral condyle fracture/nonunion with fishtail deformity of the humerus.

## Introduction

A medial condyle fracture of the humerus is very rare, especially in the adult population. Although there are a few case series describing the clinical features of this injury, two different fracture patterns might be considered: an avulsion fracture displaced downward and a compression fracture displaced upward [1,2]. In the relevant literature, there are several treatment options for medial condyle fractures, including open reduction with internal fixation (ORIF), excision of the fragment, and nonoperative treatment [1]. Although most case reports applied ORIF with Kirschner wire or screws, outcomes of the treatment reported previously varied from poor to good [1,3,4]. The optimal treatment option remains unclear, whereas surgical treatment to achieve rigid stability of the elbow joint to allow early mobilization and range of motion (ROM) exercises may be preferred.

Fishtail deformity is known to occur as a sequela of distal humerus fracture in childhood trauma, characterized as a sharp-angled wedge of the distal humerus. Regarding its potential pathogenesis, a gap in the reduction of an intracondylar fracture, avascular necrosis of the epiphysis, or central physeal arrest have been advocated [5]. The long-term outcomes in elbows with the fishtail deformity may include limited ROM, painful stiffness associated with osteoarthritis, loose body formation, cubitus valgus deformity, proximal migration of the ulna, and radial subluxation [6].

We herein report a rare case in which total elbow arthroplasty (TEA) was performed for the treatment of an elderly patient with a medial condyle fracture, preexisting fishtail deformity, and lateral condyle nonunion.

## Case presentation

**Figure 1:**
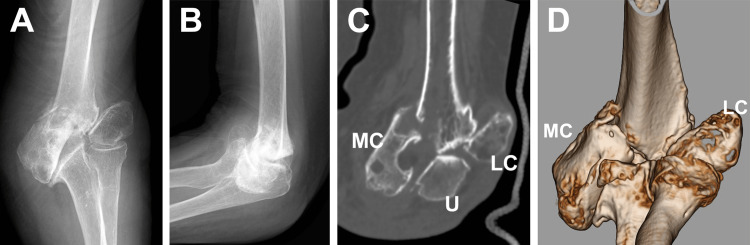
Preoperative radiographic images. The plain radiographs (A: anteroposterior image, B: lateral image) and CT images (C: coronal image, D: three-dimensional image) represent the medial condyle fracture in the presence of fishtail deformity and lateral condyle nonunion. MC, medial condyle; LC, lateral condyle; U, ulna

An 83-year-old, right-handed woman sustained a medial condyle fracture of the left elbow by falling and placing her left hand on the ground. The patient had a comorbidity of long-lasting limited elbow motion with a history of elbow trauma in childhood. The patient presented to our outpatient clinic due to severe pain around the elbow. This was preceded by conservative treatment performed at an outpatient clinic, which consisted of immobilization for four weeks in a long-arm cast. Plain radiographs and computed tomography performed at our clinic revealed unstable fracture of the medial condyle of the humerus in the presence of fishtail deformity with lateral condyle nonunion (Figures 1A-1D).

**Figure 2:**
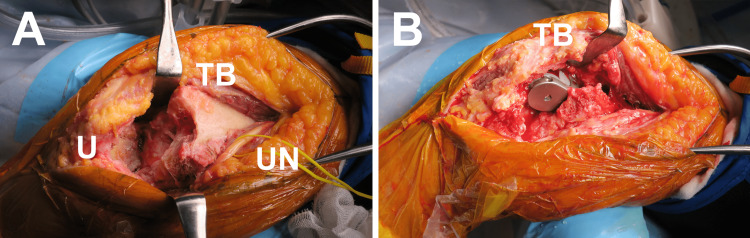
Intraoperative photographs. The surgery was performed through the triceps-on approach (A), and rigid stability at the elbow joint was achieved with semiconstrained total elbow arthroplasty (B). TB, triceps brachii muscle; U, ulna; UN, ulnar nerve

On the other hand, the patient had a good general health condition, and there was no history of chronic diseases, excepting continuous use of medications for hypertension. The patient was scheduled to undergo surgical treatment since we determined that the medial condyle fragment remained unstable with preexisting conditions and that further conservative treatment would result in nonunion. At eight weeks after the injury, the patient underwent semiconstrained TEA (Nexel Total Elbow, Zimmer Biomet Ltd., Warsaw, IN, USA). To preserve the triceps brachii muscle as well as its distal attachment, TEA was performed through the triceps-on approach [7]. After the ulnar nerve was mobilized, the triceps brachii muscle was elevated from the humerus on the medial and lateral sides, with the dissection distally between the extensor carpi ulnaris and anconeus muscles (Figure 2A). Bilateral condyle fragments and the radial head were resected for the following TEA procedure (Figure 2B), and detached musculotendinous stumps were secured to the triceps aponeurosis or remained intermuscular septum. Anterior transposition of the ulnar nerve was subsequently performed.

**Figure 3:**
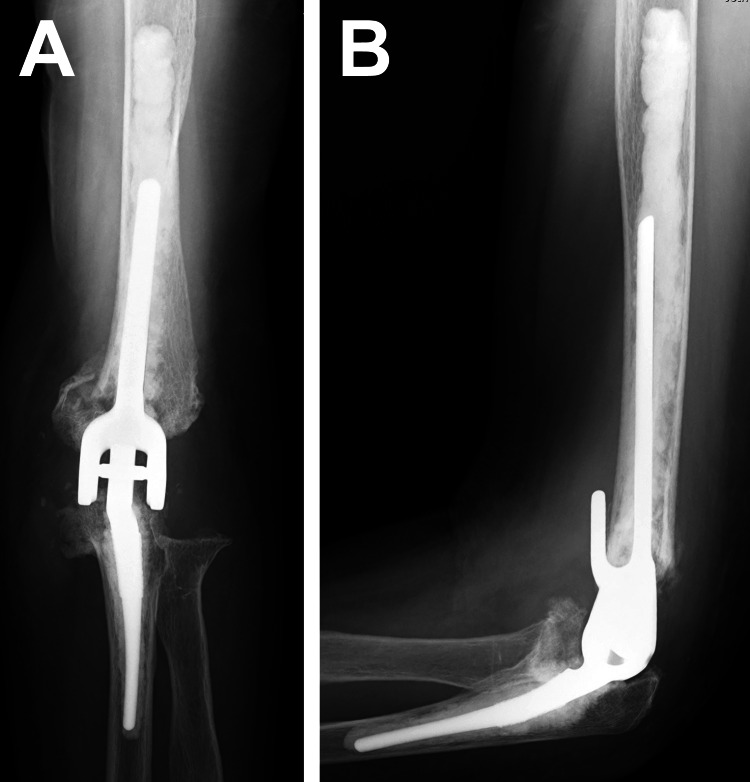
Postoperative radiographs at the one-year follow-up. The anteroposterior image (A) and the lateral image (B).

Postoperatively, the patient was immobilized with a long-arm spica orthosis for one week and underwent ROM exercises that were taught by a therapist. Daily activities were encouraged after the removal of the orthosis. The patient was instructed to avoid putting weight on the left arm and to avoid lifting more than 2.5 kilograms in weight. At the 12-month follow-up examination, the patient felt mild restriction only for elbow flexion compared to the pre-injured condition. She had no pain, and satisfactory functional outcomes had been achieved without restriction in her activities of daily living. Radiographic examination revealed sufficient stability at the elbow joint (Figures 3A, 3B).

**Figure 4:**
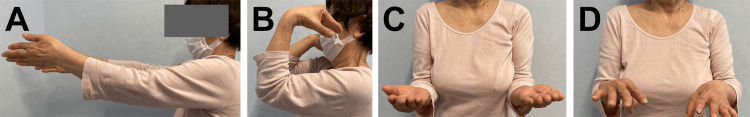
Ranges of motion at the one-year follow-up examination. Elbow extension (A) and flexion (B). Forearm supination (C) and pronation (D).

With regard to the clinical outcome at the 12-month follow-up examination, elbow ROM was -25° for extension and 115° for flexion, forearm ROM was 90° for supination and 70° for pronation, and wrist ROM was 70° for extension and 75° for flexion (Figures 4A-4D).

## Discussion

We reported the case of an elderly patient with a medial condyle fracture with preexisting fishtail deformity and lateral condyle nonunion that had been present since childhood. To date, there have been few case reports demonstrating the treatment for similar injury pattern. In addition, there have been no reports to use TEA for the medial condyle fracture excepting with elbow dislocation. For the current case, our treatment strategy using TEA to achieve rigid stability at the elbow joint resulted in satisfactory functional recovery at the one-year follow-up.

TEA has been recognized as an effective and reliable procedure for comminuted fractures of the distal humerus [8]. Although with negligible incidence of postoperative complications following TEA, several reports have confirmed satisfactory pain relief and functional recovery in elderly patients [9]. As the most significant advantage of TEA for comminuted elbow fractures, rigid stability of the elbow joint to allow early mobilization and ROM exercises should be noted.

The current case had deteriorated elbow stability due to unstable medial condyle fracture and the nonunion of the lateral condyle. A previous report showed a potential efficacy of nonoperative treatment for the medial condyle fracture [1]; however, there have been no studies supporting nonoperative treatment in the presence of preexisting fishtail deformity. Theoretically, a fishtail deformity may cause increased stress concentration at the angular wedge, and, subsequently, osteochondral lesions can be present in the capitellum [10]. According to the literature, we can assume that the base of the medial condyle in the presence of the fishtail deformity plays an essential role as the static column for maintaining elbow stability at the humerus especially with preexisting lateral condyle nonunion. As shown in the current case, upward fracture line initiating a wedge point can be associated with a compression force in elbows with fishtail deformity [5,11]. Therefore, treatment option should be carefully determined since deteriorated bipolar columns at the elbow with severe fishtail deformity may result in persistent instability. We believe that TEA may be a feasible treatment option to achieve rigid stability for the current injury.

Regarding the surgical technique, we applied the triceps-on approach to preserve the triceps brachii muscle. A recent study demonstrated the triceps-on approach has significant functional advantages over other approaches (the triceps turndown or split approaches), whereas this approach may provide a reduced exposure of the distal humerus and the proximal ulna that can affect the survivorship of TEA implants [12]. In the current case, sufficient exposure for implantation could be obtained after the resection of bilateral condyle fragments. We considered it important to secure the musculotendinous stumps detached from the condyles rigidly to the peripheral aponeurosis or intermuscular septum. In the current case, satisfactory functional recovery was obtained at the one-year follow-up; however, careful postoperative follow-up especially for the muscle strength in both elbow and wrist motion may be required.

## Conclusions

This case report described surgical treatment with TEA for a rare injury pattern comprising the medial condyle fracture with preexisting fishtail deformity and lateral condyle nonunion. The satisfactory clinical outcome in the current case may support this surgical approach to restore rigid stability for deteriorated elbow stability due to bilateral condyle fracture/nonunion with fishtail deformity of the humerus.
